# Effects of a combined strengthening, stretching and functional training program versus usual-care on gait biomechanics and foot function for diabetic neuropathy: a randomized controlled trial

**DOI:** 10.1186/1471-2474-13-36

**Published:** 2012-03-19

**Authors:** Cristina Dallemole Sartor, Ricky Watari, Anice Campos Pássaro, Andreja Paley Picon, Renata Haydée Hasue, Isabel CN Sacco

**Affiliations:** 1 Physical Therapy, Speech and Occupational Therapy Department, School of Medicine, University of São Paulo, São Paulo, SP, Brazil

**Keywords:** Biomechanics, Diabetic foot, Rehabilitation, Plantar pressure

## Abstract

**Background:**

Polyneuropathy is a complication of diabetes mellitus that has been very challenging for clinicians. It results in high public health costs and has a huge impact on patients' quality of life. Preventive interventions are still the most important approach to avoid plantar ulceration and amputation, which is the most devastating endpoint of the disease. Some therapeutic interventions improve gait quality, confidence, and quality of life; however, there is no evidence yet of an effective physical therapy treatment for recovering musculoskeletal function and foot rollover during gait that could potentially redistribute plantar pressure and reduce the risk of ulcer formation.

**Methods/Design:**

A randomised, controlled trial, with blind assessment, was designed to study the effect of a physiotherapy intervention on foot rollover during gait, range of motion, muscle strength and function of the foot and ankle, and balance confidence. The main outcome is plantar pressure during foot rollover, and the secondary outcomes are kinetic and kinematic parameters of gait, neuropathy signs and symptoms, foot and ankle range of motion and function, muscle strength, and balance confidence. The intervention is carried out for 12 weeks, twice a week, for 40-60 min each session. The follow-up period is 24 weeks from the baseline condition.

**Discussion:**

Herein, we present a more comprehensive and specific physiotherapy approach for foot and ankle function, by choosing simple tasks, focusing on recovering range of motion, strength, and functionality of the joints most impaired by diabetic polyneuropathy. In addition, this intervention aims to transfer these peripheral gains to the functional and more complex task of foot rollover during gait, in order to reduce risk of ulceration. If it shows any benefit, this protocol can be used in clinical practice and can be indicated as complementary treatment for this disease.

**Trial Registration:**

ClinicalTrials.gov Identifier: NCT01207284

## Background

### The problem of diabetic peripheral polyneuropathy

Diabetic peripheral neuropathy (DPN) is a common chronic complication of diabetes mellitus that has been very challenging for clinicians for a long time. It results in high public health costs and has a huge impact on the quality of life of patients when not treated properly. Prevention is still the most important way to avoid plantar ulceration and amputation, which is the most devastating endpoint of the disease.

More than 220 million people worldwide have diabetes [1]. DPN affects up to 50% of people with diabetes and usually starts with lesions on peripheral sensitive nerves and progresses to motor and autonomic nerves. It causes progressive loss of vibratory, thermal, tactile, and proprioceptive sensitivities, following this sequence of incidence [2]. Muscle atrophy, musculoskeletal impairments, and autonomic dysfunction can be established in later stages of the disease, mainly due to impairment of the higher diameter of neural fibers [3-5]. Although some different incapacities and comorbidities can result from DPN, common symptoms are tingling, pain, numbness, and weakness in the feet and hands [1].

The feet are the main target of most of the sensitive and motor complications to which individuals with diabetes are exposed [6-13]. Limitation of mobility of the foot and ankle joints is prevalent in patients with diabetes, as well as altered plantar pressure during gait [10]. Dysfunction of intrinsic foot muscles have been observed in patients with DPN by Boulton [14] and also can be present in diabetic patients without polyneuropathy [15]. The association among range of motion (ROM), strength, and function loss can lead to altered foot rollover during gait, as their integrity is needed to enable proper load absorption.

Biomechanical alterations in the foot rollover process during gait and its relationship with plantar ulcerations have been discussed in the literature, especially using plantar pressure distribution as an important predictor parameter [11,16,17] mainly under the forefoot [18-20]. This mechanical parameter also has been broadly used for footwear and insole prescriptions [21]. However, there are several other important biomechanical alterations in the gait of diabetic neuropathic patients that may lead to ulcer formation. These include less ankle ROM [22-24]; alterations in spatial-temporal patterns (velocity, step length, stride length, and time of double support) [9,10,25-29]; differences in kinetic patterns with modified ground reaction forces and net joint moments [25,26,30,31]; and delayed leg and thigh muscle activation [28,32-34]. Although these alterations have been identified in this population, it is still unclear if any available therapeutic interventions (pharmacological, physiotherapy, and use of orthotic devices and insoles) are efficient in restoring the biomechanical parameters to a more physiological pattern, thus reducing ulcer formation and risk of amputation.

The most common intervention for healing and minimising plantar ulceration risk is the prescription of some type of orthotic device (special footwear, casting, or insoles) to reduce plantar pressure in specific foot areas. However, a recent systematic review [35] reported that those devices are only effective in the healing process; there is still not enough evidence that they are efficient in preventing plantar ulcer formation. The prescription of this treatment is mostly based on clinical practice, but not on clear scientific evidence. There is no study available regarding prevention of a first ulcer incidence. The above-mentioned systematic review [35] reported that not all studies showed a lower ulcer recurrence in patients who used those devices; therefore, the results are still inconclusive. It is important to emphasize that this type of intervention only focuses on relieving the effects brought on by DPN that overload some plantar areas. The other impairments that are associated with these overloads, and which also may be the causes of plantar alterations (limited joint movement, muscle weakness, and sensory loss), are not the focus of prescribing orthotic devices or special shoes.

### Effectiveness of rehabilitation for DPN

#### Foot and ankle ROM improvement

Tissue alterations around distal joints, such as thickening of joint structures, tendons, and ligaments, have been clinically observed in patients [20]. These tissues contain greater quantities of collagen, and they are exposed to non-enzymatic glycosylation caused by hyperglycaemia, reducing tissue elasticity. These tissue alterations can result in foot rigidity, which in turn causes difficulties in proper segmental foot mobility and adequate foot rollover, as well as poor capacity of load absorption by the foot and ankle during daily activities [36-39]. All these changes may result in an increase in plantar pressure in subjects with diabetes [20,23,24], predisposing them to plantar ulcers [12,40]. Therefore, ROM restriction associated with a lack of protective sensation and foot deformities may even increase the force and mechanical stress exposure under the patient's foot, predisposing the foot to ulcer formation.

Specific ROM restrictions already have been shown to contribute to increased mechanical stress over the plantar surface. Rao et al. [41] showed that the smaller the first metatarsal and lateral forefoot sagittal motion and calcaneus eversion/inversion, the higher the magnitude of plantar loading under the respective segment.

The joint collagen structures respond to mechanical stresses to adapt to the types of movements and mechanical stresses that are being required [42]. Some physical therapy procedures, such as stretching and joint manipulation or mobilisation, exercise the joints, pushing them to their limits and, thus, inducing mechanical stress. Although it is not known if the remodeling of these structures is preserved in patients with diabetes mellitus, home exercise therapy has been suggested to improve distal joint mobility and plantar pressure distribution during gait in a randomised, controlled trial with DPN patients [43]. Passive and active stretching aimed at increasing ankle and first metatarsophalangeal ROM were performed for ten seconds in each joint position, up to three times a day, for one month. The results showed a decrease in peak pressure in the diabetic patients, but an increase in the control group. Although joint mobility of the ankle and the first metatarsophalangeal joint were not different between the groups after the intervention, the DPN patients showed a trend toward a decrease in joint stiffness in these segments, which could explain the decrease in peak pressure during gait. The authors justified this negative result in ROM improvement by the short duration of their intervention and the small sample size of the study (21 subjects), and they suggested further investigations with longer intervention periods.

#### Effects of DPN on foot and ankle muscles

Cross-sectional magnetic resonance images have shown that the total volume of the intrinsic foot muscles were halved in long-term diabetic patients with neuropathy, compared to diabetic patients without neuropathy and healthy non-diabetic individuals [44]. Therefore, atrophy of these muscles can be closely related to the severity of neuropathy and can reflect motor dysfunction. Although the role of foot muscle dysfunction in the etiology of foot deformities is still controversial, its association with joint rigidity represents a potential risk for plantar ulcerations [44-46]. Weakness of the intrinsic foot muscles represents an independent risk factor for plantar ulcer development [45,47], probably because it leads to an altered foot rollover during gait and, consequently, a less effective plantar load distribution [48] (Bus, 2002).

The intrinsic foot muscles are also important for maintaining the medial longitudinal plantar arch, along with plantar aponeurosis. This arch has an important role in foot dynamics during walking, providing an optimal position of the foot joints and guaranteeing a more stable lever during the push-off phase [49]. Fiolkowski et al. [50] performed a tibial nerve block, which resulted in lower activity of the intrinsic foot muscles. They observed a significant navicular bone drop after this procedure. Headlee et al. [51] induced fatigue in the same muscle group, and they also observed an important navicular bone drop. Both studies concluded that these muscles have an imperative role in supporting the longitudinal medial plantar arch, and that their weakness could contribute to a more unstable and non-functional arch.

It has been observed that the strength of the lower limb muscles can be improved through a specific muscles-strengthening program in healthy adults, by progressively increasing resistance [52]. When we consider DPN patients, the review published by White et al. [53] points out that there is not sufficient evidence to support the effects of lower limb strengthening and cardiovascular training on the improvement of their quality of life. However, all the rehabilitation protocols of this review accomplished generalised muscle strengthening, without the specificity of selecting the most impaired muscle groups due to the neuropathy: the ankle and foot intrinsic muscles. A specific strengthening program for these muscle groups (intrinsic and extrinsic foot muscles), associated with ROM improvement and functional training of foot rollover during gait, have not been studied yet.

### Balance and gait training strategies

All the motor and functional limitations caused by DPN lead to postural instability and altered locomotion biomechanics, raising the risk of falls, plantar ulcerations, and amputation of the lower limbs [29,54-56]. These progressive limitations usually worsen patients' quality of life [57]. General exercises for balance improvement have already been demonstrated to be efficient in DPN patients. Allet et al. [58,59] published two randomised, controlled trials that showed a significant improvement in time-space gait parameters, in a real-life environment, in the group that received specific training consisting of circuit training gait and balance exercises. These results showed that it is possible to improve functional and independent gait, even with sensory and motor impairments.

Interventions that focused on foot and ankle recovery and their impact on gait have presented partially good outcomes [60-62]. Some improvement in balance and confidence in a population with DPN was shown by Richardson et al. [60]. Although this study was not randomised to control and intervention groups, the patients were blinded to the treatment. The intervention was composed of a group of foot and ankle strengthening exercises in a closed kinetic chain and balance exercises in single and double support positions, performed daily for three weeks. The control group did not receive any treatment. Only the intervention group showed a significant improvement in single-leg stance time, functional reach, tandem stance time, and activities-specific balance and confidence (ABC) scores. Although muscle strength was not assessed before or after intervention, the authors discussed a possible effect in strength increase due to neural changes, which is possibly due to synchronisation of motor unit activation, rather than muscle hypertrophy. Unfortunately, there are no follow-up results after the three weeks of intervention. It is not possible to conclude that the obtained results were retained.

Gait-training strategies as an offloading technique have been studied by some authors, who reported partially good results [61,62].

In the study by Pataky et al. [61], 13 patients with DPN were trained to reduce plantar pressure while walking with instrumented insoles. They were to walk in a new self-strategy in order to perform at least seven complete steps, achieving a target of 40-80% reduction in baseline peak pressure, at specific plantar areas considered at risk for ulceration. The training period was approximately one hour for only one day. A significant reduction of peak pressure was observed at the retention periods (after 30 min, one day, five days, and ten days), although the peak pressure increased at the end of the ten days. However, the patients who were trained did not have severe neuropathy, with foot deformities or joint rigidity, factors that are well recognised as being at risk for foot ulceration. Another limitation of this study was that there was no control group.

In the randomised, controlled trial conducted by York et al. [62], DPN patients were trained to walk to reduce plantar pressures using the hip gait strategy, discussed by Mueller et al. [25]. Both groups of patients were instructed to walk, with instrumented insoles, pulling their legs forward from the hip to initiate the swing phase rather than pushing off the ground with the forefoot, assuming that using the calf muscles to propel the body forward would increase the pressure under the forefoot. The control group did not have the plantar pressure biofeedback. The training period consisted of only two days, with ten trials of gait practice each day. The retention periods were at the end of day one, end of day two, and after one week of practicing at home. The results showed no reduction in peak pressure in the foot areas, and no retention periods. Although the randomised, controlled study of York et al. [62] used a similar training volume as the study of Pataky et al. [61], the use of the hip gait strategy in order to minimise the pressure under the forefoot did not seem to reduce peak pressure in the foot areas, even with the help of instrumented insoles. The authors pointed to some limitations of this study, including the short training period and possible inclusion of non-neuropathic patients in the intervention group.

Both studies, by York et al. [63] and Pataky et al. [61], have the same limitations: a short training period and a short retention period. In addition, they had only trained the walking task, without any exercise for ROM gain or muscle strengthening of the foot and ankle complex. Perhaps the success of their intervention would depend on the association of these aspects with gait training as well.

### Hypotheses

The literature update shows that, with ROM exercises and gait training, individuals with DPN can improve their confidence and balance, and they can change plantar pressure distribution during gait, although retention of these interventions was not studied for more than ten days. There is still a gap in the literature regarding whether a specific training for improving foot rollover and redistributing plantar pressure during gait could be effective in predicting risk of ulceration over a long-term period. If we look at some of the interventions applied to this population, general lower limb exercises of the ankle, knee, and hip are recommended, without training the small foot joints, which play an important role in foot rollover and load absorption during locomotion. Conversely, other studies only applied segmental exercises, without training foot rollover during gait. None of the therapies cited were able to integrate the peripheral gains (muscle strength and ROM improvements) to foot function during daily locomotor tasks.

Considering the possibility of recovering some foot and ankle function, foot rollover could also be improved, and consequently, the risk of foot ulceration could be reduced. This approach is quite new, as the standard treatment has focused on plantar load relief by introducing orthotic devices and insoles that passively change the plantar pressure distribution, without exploring the possibility of improving function. That is, this new intervention focuses on recovering the causes of movement abnormalities, intending a redistribution of plantar pressure during gait through an active intervention. Although some of the impairments are permanent, such as sensitivity loss, recent evidence leads us to believe that it is possible to attain some recovery.

A well-designed therapeutic exercise protocol, including interventions aimed at joint, muscle, and locomotor task recovery, should be part of a physical therapy routine prescription. Although this comprehensive approach is imperative in clinical practice, its functional and biomechanical effects have not yet been tested. Most importantly, if effective, these exercises might be performed by the patient without needing physiotherapy supervision, representing a way of reducing costs associated with diabetic patients' treatment.

Our hypotheses are:

(I) The specific training proposed could improve muscle function and foot and ankle ROM.

(II) It could be reflected in the foot rollover during gait, improving plantar pressure distribution and lower limb kinematics.

Therefore, our aim is to investigate the effect of exercise therapy intervention on foot rollover during gait, ROM, and muscle strength and function of the foot and ankle complex, as well as balance confidence when performing walking tasks.

## Methods/Design

### Overview of research design

A randomised controlled trial was designed to study the effects of the intervention. Patients diagnosed with DPN are recruited from the University hospital and referred to a physiotherapist, who performs the initial blind assessment.

**Figure 1:**
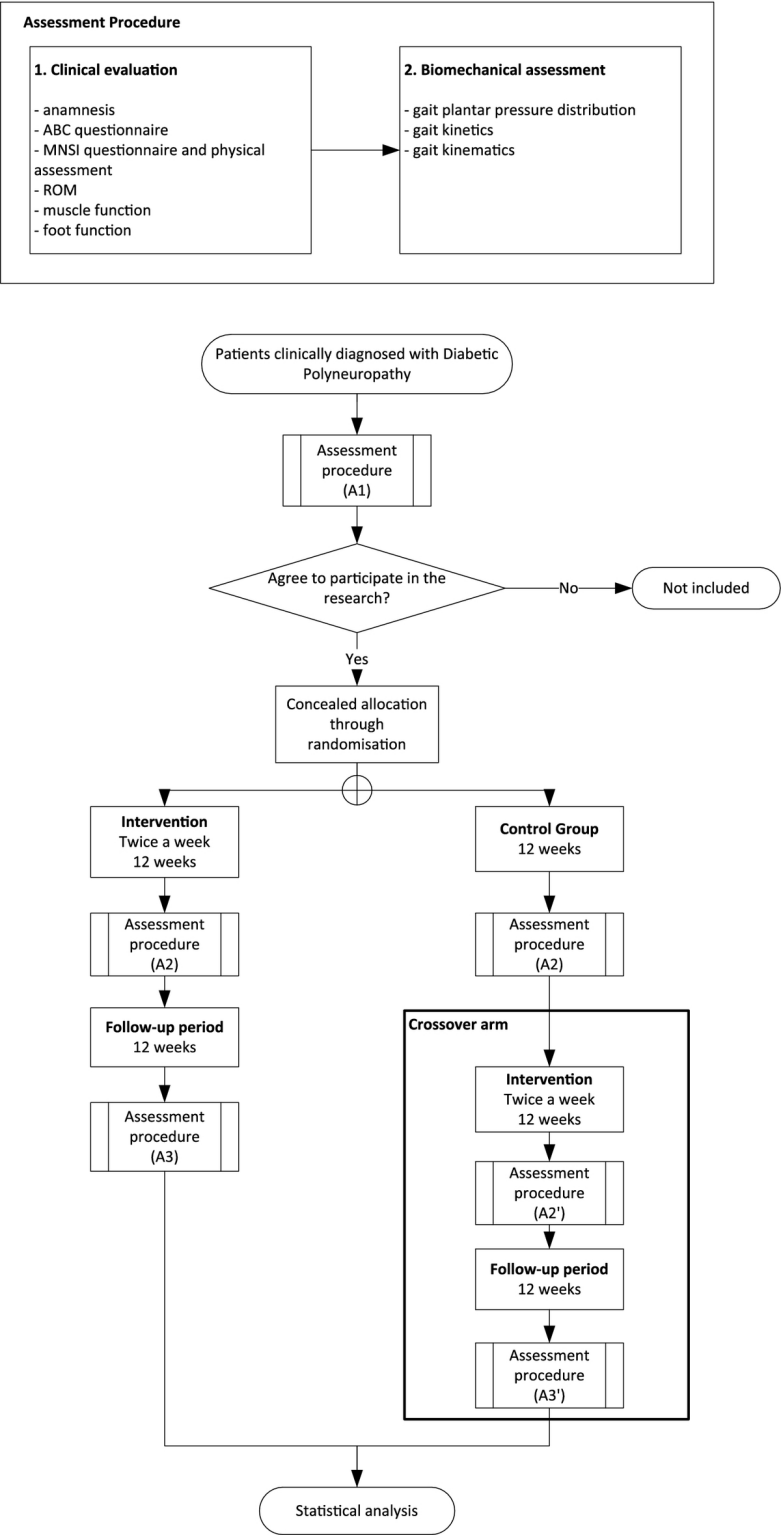
**Flowchart of the protocol steps**.

The design and flowchart of the steps of the protocol are presented in Figure 1.

The patients allocated to the intervention group receive the treatment for 12 weeks, twice a week, 40-60 min per session. They are assessed at baseline condition (A1), after 12 weeks (A2-intervention period), and after 24 weeks (A3-follow-up period).

The patients allocated to the control group represent the crossover arm of the study. They also are assessed at baseline condition (A1) and after 12 weeks (A2). During this period, they continue to receive the usual recommended medical care at the hospital, which includes pharmacological treatment and self-care instructions. After the end of the second evaluation, they receive physiotherapy intervention for 12 weeks and are then assessed after intervention (A2'-24 weeks after baseline) and after the follow-up period (A3'-36 weeks after baseline). This design was chosen in order to increase the number of patients receiving the intervention, as we can expect losses to the follow-up evaluation due to the longer period of time required to complete all the protocol procedures (intervention and follow-up period).

### Participants and recruitment

This study is currently recruiting patients (study start date: August 2010).

The eligibility criteria are:

- patients 45 to 65 years of age

- diabetes mellitus type 1 or 2, diagnosed for at least seven years

- body mass index ranging between 18.5 and 29.9 kg/m^2 ^(normal and overweight groups)

- presence of DPN previously diagnosed by the medical care centre

- score higher than 2 out of 13 in the questionnaire of the Michigan Neuropathy Screening Instrument [64,65], indicating the presence of at least two DPN symptoms

_- _score higher than 1 out of 10 for physical assessment of the same instrument, but always including at least impaired vibration perception

- ability to walk independently in the laboratory space

- any plantar ulceration should be healed for at least six months

- not having partial or total foot amputation

- not receiving any physical therapy intervention

Patients are not selected if they have other neurological or orthopaedic impairments (such as stroke, cerebral palsy, poliomyelitis, rheumatoid arthritis, prosthesis, or moderate or severe osteoarthritis), major vascular complications (venous or arterial ulcers), severe retinopathy, or severe nephropathy that causes edema or requires haemodialysis.

The participants are recruited from three settings: (a) diabetes mellitus ambulatory medical care located in a regional hospital, (b) National Association of Diabetes Mellitus (ANAD), and (c) patients from a primary care centre at the School of Medicine of the University. The potential patients are interviewed by telephone and, when selected, are assessed in the laboratory to confirm all the eligible criteria. This first laboratory assessment represents the baseline condition (blind assessment).

The patients allocated to the intervention group are treated in the Physical Therapy Department, in an ambulatory setting that assists all the physical therapy treatments of the Department, providing a real environment for the intervention.

### Randomisation and blinding

The randomisation schedule was prepared by an independent researcher who was not aware of the numeric code for the control and intervention groups, using Clinstat software [66]. A numeric block randomisation sequence is kept in opaque envelopes.

After the patients' agreement to participate in the research, the allocation into the groups is made by another independent researcher, who is also unaware of the codes. Only the physiotherapist knows who is receiving the intervention.

### Clinical assessment

All the assessments are performed by a physiotherapist who is blind to group allocation of the patients. Each assessment consists of anamnesis for personal details, diabetes history, and any other health issue of interest. The Michigan Neuropathy Screening Instrument questionnaire and physical assessment are used [64,65] to characterise the signs and symptoms, and to monitor the disease status.

To assess the patients' confidence in performing daily locomotor skills, we chose to use the Activities-Specific Balance Confidence Scale (ABC) [67].

We also measure passive and active ankle ROM and first metatarsophalangeal joints in the sagittal plane. For the ankle measurement, we use an electrogoniometer (model SG110/A and SG 150, Biometrics, Gwent, England). For measuring the first metatarsophalangeal joint, we use a manual goniometer.

Intrinsic and extrinsic foot and ankle muscle functions are assessed through manual testing [68], as there is not currently an instrument available that is capable of measuring the function of this group of muscles. Other authors also use manual testing and consider it a useful tool [45]. The assessed muscles are triceps surae, tibialis anterior, interosseous, lumbrical, flexor hallucis brevis, flexor digitorum brevis, extensor hallucis longus and brevis, and extensor digitorum longus and brevis.

Foot function is assessed based on a test protocol designed by Palmer and Epler [69], which consists of asking the patient to perform, as quickly as possible, the following tasks, while seated: (a) grab a cotton piece with the toes, keeping the heel on the floor, and (b) raise only the toes, keeping the heel and forefoot on the floor. Then, in a standing position, the patient is asked to (a) raise the forefoot and (b) raise the heel. These tests are very practical and easily reproducible. Each test has a scale relating to the number of movement repetitions: 'absent functionality' (zero repetitions), 'little functionality' (1 to 4 repetitions), 'reasonable functionality' (5 to 9 repetitions), and 'normal functionality' (10 to 15 repetitions).

### Biomechanical assessment

Plantar pressure is recorded using the Pedar-X system (Novel, Munich, Germany) at 100 Hz. The patient walks barefoot on a 10 m flat walkway at a self-selected cadence (controlled between subject's trials within 96-116 steps/min), with the insole placed and fixed, using an anti-skid sock and a stripe at the ankle. Four valid trials are recorded, and we discard the first and last steps from the analysis. The foot is divided into six areas (heel, midfoot, lateral forefoot, middle forefoot, medial forefoot, hallux, and toes), using the same software for data acquisition. A time-series analysis will be performed to compare the pressure curve in each area, over the stance duration, intending to describe changes in plantar pressure distribution. Values of contact area and peak pressure will be compared in these six areas.

Kinematic gait parameters are acquired using three-dimensional displacement of passive reflective markers (20 mm in diameter) tracked with six infrared cameras (OptiTrack FLEX: V100, Natural Point, Corvallis, OR, USA) (Trombini-Souza et al., 2011). The markers are placed on the subject using a standard Cleveland Clinic marker set (iliac spine antero-superior, superior aspect of the greater trochanter, lateral knee joint line, lateral malleolus, calcaneus, and head of the fifth metatarsal) [70]. Extra markers are placed bilaterally at the medial knee joint line, medial malleolus, and first metatarsal joint for the static standing trial, in order to determine relative joint centres of rotation for the knee, ankle, and longitudinal axis of the foot. These extra markers are removed in the gait trial. In addition, three non-collinear reflective markers are fixed at two squares, forming sets of technique cluster. One of these is placed in the lateral thigh and the other over the shank. Theses landmarks are determined by the same physiotherapist who performs the blind assessment. The laboratory coordinate system is established at one corner of the force plate, and all initial calculations are based on this coordinate system. Each lower limb segment (foot, shank, and thigh), based on surface markers, is modelled as a rigid body with a local coordinate system that coincides with the anatomical axes, and translations and rotations of each segment are reported relative to neutral positions defined during the initial standing static trial.

Ground reaction forces are acquired by a force plate (AMTI OR-6-1000, Watertown, MA, USA) embedded in the centre of the walkway.

Force and kinematic data acquisition are synchronized and sampled by an A/D card (AMTI, DT 3002, 12 bits) at 100 Hz. Mathematical analysis of the kinematic data will be performed using Visual3D software (C-motion, Kingston, ON, Canada), and the ground reaction force analysis will be performed using a custom-written Matlab function (MathWorks, Natick, MA, USA). The variables to be analysed are: (1) joint angles and (2) net ankle moments in the sagittal and frontal planes; and (3) step length and (4) duration. These variables will contribute to discussion of the possible changes in plantar pressure distribution, especially the ankle position in the initial and terminal stance phases.

### Outcome measures

The main outcome measure is foot rollover, which will be described by time-series analysis of plantar pressure distribution during gait. This variable was chosen because it reflects the alterations of kinematics, kinetics, and muscle function in the dynamic task of gait.

The secondary outcomes are foot and ankle kinetics and kinematics during gait, neuropathy signs and symptoms, foot and ankle ROM, function and muscle strength, and Activities-Specific Balance Confidence Scale [67].

### Intervention rationale

This intervention protocol is based on evidence that shows that:

(1) Foot rigidity is associated with increasing local loads and predisposes to plantar ulceration [12,20,40]. The increase in ROM of these segments could contribute to restoring foot rollover during gait.

(2) The weakness of the intrinsic foot muscles and ankle flexors and extensors represents an independent risk factor for the development of plantar ulcers, leading to a less effective plantar load distribution [14,45,47]. The strengthening and recovery of their function also could be reflected in foot rollover during gait.

(3) There is evidence that shows that patients with DPN can improve gait and confidence, suggesting a possible recovery of motor control functions at some level [58-60]. A more comprehensive exercise therapy should integrate the peripheral gains (increase in ROM and muscle function and strength) into motor tasks, such as gait. It could be achieved when requiring these gains during the execution of walking skills and simple balance exercises.

(4) The patients should perform the exercises independently at home, and the exercises should be simple enough to allow that.

The complete description of the intervention can be found in Additional file 1: Table S1.

We divided the therapeutic sessions into four blocks of exercises, characterised by the main objective of each exercise group. They are: (a) gain of foot and ankle ROM, (b) foot and ankle muscle strengthening, (c) foot and ankle functional exercises, and (d) walking skills and foot rollover training. Each session is composed of some of the exercises from the four groups. Gradual and progressive difficulty is offered to the patient, respecting any limitation due to pain and/or decrease in performance during execution. In addition, in each session, the exercises are performed following an order that starts with the passive exercises, progresses to active, and finishes with walking and functional skills. Therefore, we can promote the motor integration of peripheral gains into functional movements in every session. Although this intervention is focused only on foot and ankle exercises, we have a complete approach to the rehabilitation process that depends on the association between the afferent and efferent peripheral system and the central system to perform tasks of daily living, such as walking. Previous studies do not accomplish that specificity of selecting the segments most impaired by DPN to recover, nor do they integrate the musculoskeletal gains in the foot rollover process during gait.

During all exercises, the physiotherapist focuses on proper alignment of the segments, especially if the patient has difficulty in maintaining it, in a way that no movement compensations are allowed. During weight-bearing exercises, additional care is taken to maintain proper foot support: the toes should always touch the floor, avoiding hammering or clawing when possible, and the ankle should not be laterally tilted (with lateral deviations). Thus, self-perception of the foot and ankle position is stimulated even during the most challenging tasks.

The discontinuation criteria for the exercises during one session are cramps, moderate to intense pain, fatigue, dizziness, fear, or any other condition that exposes the patient to any kind of risk or discomfort.

### Sample size and statistical analysis

The sample size calculation was made using an effect size of 0.36 (moderate effect size), considering the primary outcome measure of peak plantar pressure. We took the SD estimates from a study we completed, wherein we recruited a similar patient cohort [71]. A sample size of 46 subjects is needed to provide 81% power to detect a moderate effect difference between the highest and lowest group pressure means, assuming an alpha level of 0.05 and an statistical design of F test of repeated measures (between and within effects), and assuming a 10% loss to follow-up. The statistical analysis will be based on intention-to-treat analysis, and general linear models of analysis of variance for repeated measure will be used to detect treatment-time interactions. The outcome measures will be compared among baseline, 12 weeks, and 24 weeks. Cross-correlation analysis will also be provided between primary and secondary variables if it shows any relevance.

### Ethics and data security

This trial was approved by the Ethics Committee of the School of Medicine of the University of São Paulo (Protocol number 054/10). All the patients will be asked for written informed consent according to the standard forms.

## Discussion

We have presented the problem of DPN and its current interventions for changing gait strategies. We also have presented a new and comprehensive intervention protocol aimed at recovering foot and ankle function and enhancing their ROM. This approach intends to recover at least some of the specific deficits caused by DPN and to promote the motor integration of peripheral gains into foot rollover during gait, redistributing plantar pressures in this task. These deficits are directly related to abnormal foot pressure during gait, which is also related to plantar ulcer incidence. If this partial recovery shows to be possible, such as reducing plantar pressures and increasing contact areas, we can assume that ulcer incidence can be influenced by it at some level.

## Competing interests

The authors affirm that this study has not received any funding/assistance from a commercial organization and we do not keep any commercial relationships which may lead to a conflict of interests.

## Authors' contributions

ICNS, CDS, RW, RHH are responsible for designing the study. ICNS, CDS, ACP and APP act as trial coordinators. All the authors have approved the final manuscript.

## Pre-publication history

The pre-publication history for this paper can be accessed here:

http://www.biomedcentral.com/1471-2474/13/36/prepub

## Supplementary Material

Additional file 1 **Table S1**. Description, execution, and progression parameters of the exercises included in the intervention protocol. Click here for file
