# Supplementary material for: Effects of a combined strengthening, stretching and functional training program versus usual-care on gait biomechanics and foot function for diabetic neuropathy: a randomized controlled trial
Source: BMC Musculoskelet Disord. 2012 Mar 19;13:36. doi: 10.1186/1471-2474-13-36 (PMC3395854; doi:10.1186/1471-2474-13-36)
Supplement: Additional file 1 — Table S1. Description, execution, and progression parameters of the exercises included in the intervention protocol. [file 1471-2474-13-36-S1.PDF]

Table 1. Description, execution, and progression parameters of the exercises included in the intervention protocol.

	SEGMENTAR EXERCISES		MOTOR AND SENSORIAL INTEGRATION	
**Exercises**	**ROM improvement**	**Muscle strengthening**	**Balance training**	**Gait training**
**Description**	1. Passive stretching of flexors and extensors of toes and hallux (Goldsmith et al., 2002).<br>2. Self-stretching of triceps surae (Goldsmith et al., 2002).	1. Hallux and toe flexor muscles, and foot intrinsic muscles.<br>2. Hallux and toe extensor muscles.<br>3. Flexors, extensors, and inversor and eversor muscles of the foot and ankle complex (Richardson et al., 2001; Gardner et al., 2001).	1. Single-leg support (Richardson et al., 2001).<br>2. Double-leg support on a rubber disc filled with air.	1. Walking over the heel, forefoot, lateral border, and medial border of feet (Gardner et al., 2001) .<br>2. Walking in tandem Gardner et al., 2001).<br>3. Walking, softening the heel and forefoot contact during normal walking.<br>4. Walking, grabbing the floor with toes.<br>5. Walking with the considered normal foot rollover: heel strike, midfoot, lateral forefoot, medial forefoot, and hallux contact.
**Execution**	1. Supine, knees extended, ankle in neutral. Flexion and extension of the toes and hallux, separately.<br>2. Standing the contralateral limb ahead, with bent knee, and the ipsilateral backwards with the foot facing forward.	1. Sitting with the foot flat on the floor, grab an object with the toes and hallux.<br>2. Sitting with the foot flat, extension of the toes and hallux. No dorsiflexion allowed.<br>3.a. Supine with the knees extended, flexion, inversion, and eversion against the resistance of a rubber band.<br>3.b. Standing, flexion and extension of both ankles. Upper limbs support.	1. Standing without upper limb support.<br>2. Trying to stand still, without upper limbs support.	1, 2, 4 and 5. Walking in a straight line, with the preferred speed.<br>3. Walking in a straight line, with the preferred speed. Instruction to patient: “don´t make noise when your foot lands, and walk at your preferred speed”.
**Volume and duration**	5 X 30 seconds each limb	1. 3x of 30 repetitions.<br>2. 3 x 30 repetitions.<br>3.a. 3 x 15 repetitions.<br>3.b. 3 x 15 repetitions.	1. 5 x 30 seconds each side.<br>2. 1 x 2 minutes.	Walk for 60 meters each task.
**Progression**	Until reaching full ROM	1. Increasing the rigidity of the object: cotton, soft sponge, soft rubber ball, hard rubber ball, and pencil.<br>2. No increase in external resistance.<br>3.a. Progressive resistance of Theraband: red (medium) and black (special heavy).<br>3.b. Initially double-leg support, progressing to single-leg support.	1. Stable floor, unstable floor (folded towel) and eyes closed.<br>2. Eyes open and eyes closed.	Until performing 60 meters of each tasks, without needing intercalation.
**Progression parameters**	Without pain	1. Always perform the complete number and duration before progressing to the more difficult object.<br>2. Performing the exercise without pain or fatigue.<br>3. Always try to do the three series, even if the patient cannot reach 15 repetitions.	Maintaining the position without upper limb support.	Performing the task with proper alignment, without intercalation, and without pain or fatigue. If necessary, the patient can intercalate tasks until he is able to perform the required 60 meters.
**Approximate duration**	20 minutes	15 minutes	7 minutes	15 minutes
